# Research on Salt Corrosion Resistance of Lithium-Based Protective Coating on Mortar Substrate

**DOI:** 10.3390/ma16093463

**Published:** 2023-04-29

**Authors:** Jiawei Zang, Chonggen Pan, Xu Li, Keyu Chen, Danting Chen

**Affiliations:** 1 Department of Structural Engineering, College of Civil Engineering and Architecture, Zhejiang University, No. 866 Yuhangtang Road, Xihu, Hangzhou 311400, China; 2 Department of Civil Engineering, School of Civil Engineering & Architecture, NingboTech University, No. 1 Qianhu South Road, Yinzhou, Ningbo 315100, China; 3 Department of Civil, Environmental and Geomatic Engineering, University College London, Gower Street, London WC1E 6BT, UK

**Keywords:** nano-waterproof modification, sodium salt, durability, chloride ion erosion

## Abstract

The present study concerns hydrophobic surface treatments with silane-based coating on concrete surfaces against external ionic transport. The nano-modification and organic–inorganic modification were carried out on it and applied to the mortar matrix and concrete matrix. Lithium-based protective coating (PC1, PC2), nano-modified coating (NC1, NC2) and organic–inorganic composite coating (OL1) were prepared. The salt erosion resistance of the mortar matrix and concrete matrix was tested, compared with the blank group and the market. The test results found that the organic–inorganic modified OL1 and LC1 coatings have the greatest influence on the chloride penetration resistance of the mortar matrix, in which the chloride penetration depth of 28 days is reduced by 73.03% and 63.83%, respectively, compared with the blank group. The rate of mass change of the blank group, PC1 and PC2 coatings, and NL1 and NL2 coatings were 0.17%, 0.08%, and 0.03%, respectively. The result demonstrated that the lithium-based coating could effectively delay the penetration rate of chloride ions and sulfates into the mortar, and the nano-modified properties could improve the salt resistance. The scanning electron microscopy (SEM) showed that coating treatment would promote the secondary hydration of cement-based materials, by reducing the content of $\mathrm{Ca}{\left(\mathrm{OH}\right)}_{2}$ in hydration products of cement-based materials and producing C-S-H gel, which is conducive to strength enhancement and pore refinement. The nano-component would promote the reaction degree, while the organic–inorganic coating would have the respective advantages of the two components.

## 1. Introduction

The invention of hydraulic cement has enabled concrete to become an indispensable building material, and it has become the most widely used in modern civil engineering [1]. However, the inadequate durability and solidity of the concrete have drawn the attention of many scholars and engineers within the wide application of concrete structures. As a result of neglecting the risk of lacking durability of the concrete, economic losses and safety accidents have seriously increased. Wu (2011) reported a highly annual loss of 500 billion yuan because of concrete corrosion. For example, aging diseases have become increasingly prominent due to the prolonging service life of concrete buildings, especially the surface performance of concrete. Physical and chemical erosion are the main processes responsible for deteriorating the performance of cement-based materials. Regarding physical erosion, environmental effects (e.g., erosion of wind, sand and physical abrasion) accelerate the degradation of the performance of the concrete structure. For chemical corrosion, concrete carbonization and chloride corrosion could aggravate the cracking and even the falling-off of the reinforced protective layer, causing serious harm to the concrete structure [2,3]. The current main protective measures include mixing various mineral admixtures, an anti-corrosion coating of reinforced bars, a corrosion inhibitor, an anti-corrosion coating on the concrete surface, etc. [4,5,6]. As a more economic and effective measure [7,8], concrete surface protection treatment has become a hot research area in China [9].

Organic materials are the most commonly used for surface protection, and accordingly, the most common organic surface treatment agents include silane, siloxane, epoxy resin, acrylic resin, polystyrene resin, etc. [10,11,12,13,14]. Organic surface protection materials have good hydrophobicity, and they can form a hydrophobic film on the surface of concrete and block the pores [15,16,17,18]. However, some disadvantages are present, such as poor fire resistance, hardness of cleaning, and aging easily under long-term high temperatures and ultraviolet radiation. Aging could consequently bring performance degradation, resulting in cracks or spalling [19,20]. In contrast, inorganic surface protective materials have better anti-aging properties. Inorganic surface treatment agents can penetrate the concrete and cause complex physical and chemical reactions with cement hydration products, forming new materials to plug capillary pores, increasing the surface compactness of the concrete, and reducing the surface shedding caused by load and abrasion. Thus, it could effectively prevent corrosive substances from entering the external environment of the concrete, protecting steel bars from corrosion, improving the durability of reinforced concrete structures [21].

Silicate is an excellent material of inorganic protective coating. The research into inorganic protective coating on silicate concrete surfaces began in the 1990s. Mcgettigan et al. [22] proposed that sodium silicate and calcium hydroxide in concrete would react and form precipitated silica, which would plug the pores and reduce the air as well as water permeability coefficient of the concrete surface. Studies by Thompson et al. [23] showed that sodium silicate could react with calcium hydroxide on the concrete surface by forming calcium silicate gel, which fills the surface pores and improves the durability of concrete. Based on the worldwide research, the solidifying agent of sodium silicate hardly improves the durability of concrete, especially regarding the resistance to chloride ion penetration and carbonation. Lithium silicate and nanometer materials are seldomly used as the protective coating of concrete, and only a few studies focus on the effect of coating on the surface properties and microstructure of concrete. Several scholars’ experiments have shown that nanomaterials can enhance coatings’ strength, hardness, and ductility. According to Zhao et al.’s research [17], ${\mathrm{TiO}}_{2}$ nanoparticles can increase the polymer coatings’ elongation at break due to the induction of superficial functional groups on nanoparticles and the modification of the bonding interface’s defect structure. Moreover, Li [24] characterized the microstructure of cement treated with ${\mathrm{SiO}}_{2}$-modified geopolymer coating and discovered that ${\mathrm{SiO}}_{2}$ nanoparticles act as nucleation sites that react with calcium hydroxide to create a dense C-S-H structure. By enhancing the composite materials’ performance on a molecular scale, organic–inorganic modified composite coatings can provide several benefits such as low gas permeability and high flame resistance [25,26,27]. Epoxy resins are often used as matrix materials for nanocomposite coatings in concrete protection because of their excellent adhesion and tightness properties [28,29]. For instance, Habib et al. [30] developed epoxy nanocomposite coatings by incorporating zirconia nanoparticles, leading to superior corrosion protection in 3.5 wt% NaCl solution. Incorporating nano-${\mathrm{SiO}}_{2}$ into epoxy coatings can enhance the long-term carbonation resistance of concrete, increasing the average service life of the coatings by 78% [31].

To sum up, some scholars have carried out some research on nano-modified coatings and organic–inorganic modified coatings, but little has been investigated about the chloride corrosion resistance, carbonation resistance and action mechanism of ${\mathrm{Li}}_{2}{\mathrm{SiO}}_{3}$ coating in concrete. The durability of the coating needs to be further improved. Therefore, it is necessary to further carry out the nano-modification of coating components and the waterproof modification of coating. In addition, it is a reasonable research direction to carry out surface organic–inorganic modification and nano-modification research aiming at the problems of inorganic coating of concrete, so that the combination of inorganic and organic surface protection technology could complement each other, leading to a better effect. In this paper, therefore, a nano-modified as well as organic–inorganic modification permeable silicate-based protective coating was prepared, and salt corrosion resistance of the mortar and concrete substrate after coating was tested. Scanning electron microscopy (SEM) and X-ray diffraction (XRD) were applied to analyze the chemical reaction of the silicate in concrete and the concrete mechanism of salt erosion resistance.

## 2. Materials and Method

### 2.1. Raw Materials

(1) Lithium silicate

Sodium silicate solution with a modulus of 4.8 and solid content of 23% was used. It is an odorless, tasteless, alkalescent (pH value is about 11) and transparent liquid with a relative density of 1.16~1.19 g/mL. It is soluble in water and alkaline solvent, but insoluble in alcohol and organic solvents. After evaporation, a dry film could form, which is water-irreversible.

(2) Sodium silicate

Sodium silicate (Na_2_OmSiO_2_) with a modulus of 3.3, solid content of Na_2_O of 8.3%, solid content of SiO_2_ of 36.5%, and pH of 11~12 was used, which is a transparent viscous liquid.

(3) Sodium methylsilicate

Sodium methylsilicate (CH_5_SiO_3_Na), known as a silicone waterproof agent, with a pH of 13 and proportion of about 1.1~1.3, was used. Sodium methylsilicate could react with water and carbon dioxide to form methylsilanol, which could further react with cement-based material to form an insoluble waterproof polymer compound film on its surface.

(4) Nano silica sol

Nano silica sol is the dispersion of nano-sized SiO_2_ particles in an aqueous solution, and its molecular formula is mSiO_2_·nH_2_O, in which the content of SiO_2_ is 30 ± 1% and the content of Na_2_O is less than 0.4%. The pH value is 9~10.5, the density is 1.19~1.21 g/cm^3^, and the particle size of nano-SiO_2_ is 10~20 nm. Nano silica sol (mSiO_2_·nH_2_O), which is the dispersion of nano-sized SiO_2_ particles in an aqueous solution, with the content of SiO_2_ of 30 ± 1% and Na_2_O less than 0.04%, pH of 9~10.5, density of 1.19~1.21 g/cm^3^ and a particle size of nano-SiO_2_ of 10~20 nm was used.

(5) Silane coupling agent

KH560 silane coupling agent (γ-Epoxy propoxy propyl trimethoxysilane) was used, whose molecular formula is C_9_H_20_O_5_Si. It is a colorless transparent liquid, and its concentration is 97%.

(6) Cement

**Figure 1:**
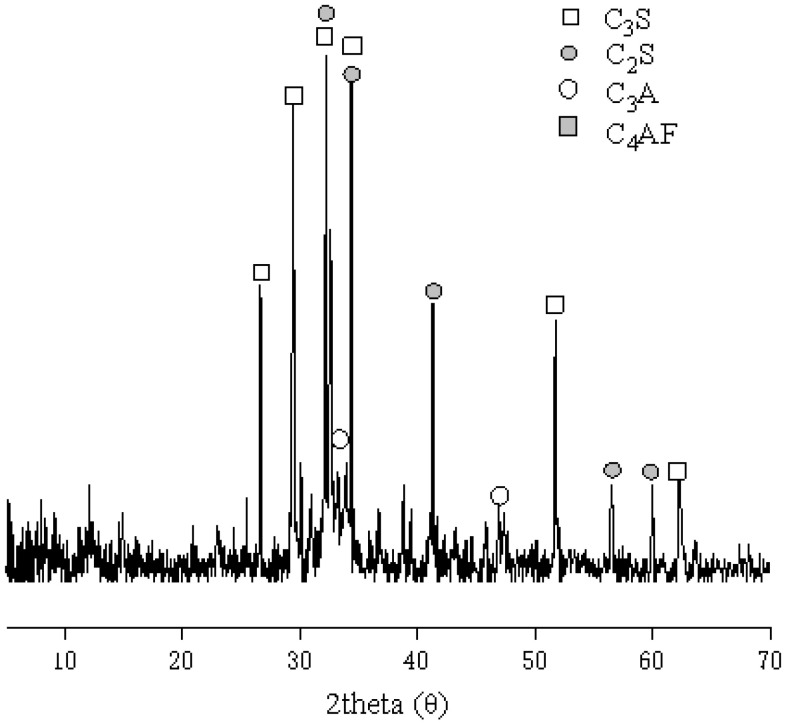
XRD spectrum of Portland cement used in the experiment.

P·O 42.5 ordinary Portland cement produced by Zhejiang Qianchao Holding Co., Ltd., Hangzhou, China, was used in this experiment, of which the basic performance index and chemical composition are shown in Table 1 and Table 2, and the XRD pattern is shown in Figure 1.

(7) Sand and stones

The sand used in the mortar test is ISO standard sand of China (Table 3). International standard organization (ISO) sand with a specific gravity of 2.64, water absorption of 1.40%, and fineness modulus of 2.68 was used as the fine aggregate.

### 2.2. Experimental Programme

In this study, the optimal mix proportion of several coatings was determined by orthogonal experiments in the research of Li Xu [24], and the coating was applied to the surface of the mortar substrate. Furthermore, these coatings were compared with a lithium-based coating (LC1) on the market, and the salt corrosion resistance of mortar substrate samples after curing for 7 days was studied. The formulations of several coatings are shown in Table 1, including lithium-based protective coatings (PC1, PC2), nano-modified coatings (NC1, NC2) and organic–inorganic composite coatings (OL1). The ratio of each coating sample is present in Table 4.

#### 2.2.1. Penetration Depth of Free Chloride Ion in a Coated Mortar Matrix

The coating samples and blank control samples were continuously immersed in sodium chloride solution for 28 days, and the free chloride ion penetration depth was measured, respectively, after immersion for 3 d, 7 d, 14 d, 21 d and 28 d.

**Figure 2:**
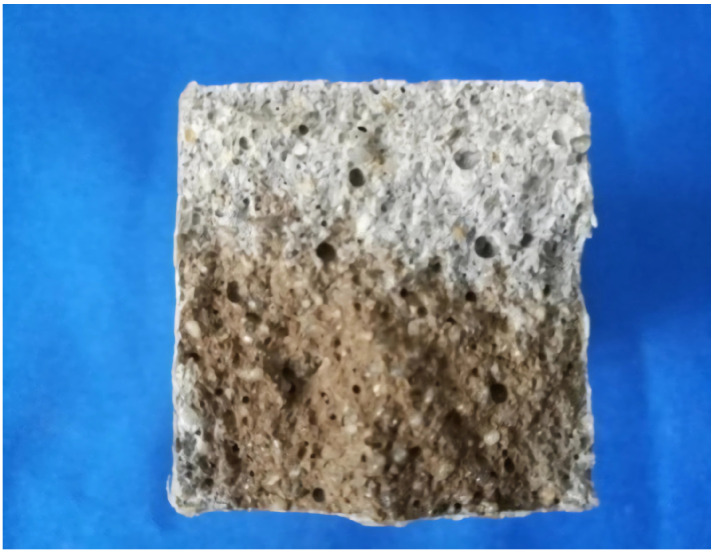
Chloride ion penetration area.

The penetration depth of a free chloride ion of the mortar matrix in a 3.5% sodium chloride solution was used as the experimental method to evaluate the resistance to chloride ion penetration (Figure 2).

The chloride ion penetration resistance test of concrete was carried out according to the electric flux method in Chapter 7 of GB/t50082-2009, “test methods for long term performance and durability of ordinary concrete”, and the electric flux of concrete coated with protective coating was determined. The specimen was a circular cylinder with a diameter of 100 mm and a height of 50 mm. The specific operation was carried out according to the following steps:

(1) The cured 28-day-old specimens were taken out, and the sides of the cylinder were sealed with epoxy resin sealing material after the surface was dry, and then we performed vacuum saturation treatment on the specimens.

(2) The test blocks were installed and their tightness was checked. Then, a 3.0% NaCl solution and 0.3 mol/L NaOH solution was injected into the experimental tanks on both sides, respectively. The negative electrode was connected with a copper mesh in the NaCl solution and the positive electrode was connected with a copper mesh in the NaOH solution.

(3) A 60 (±0.1) V DC constant voltage was applied between the two copper grids. We recorded the current value every 30 min for 6 h. The initial current value was recorded as $I_{0}$.

(4) The electric flux of each specimen could be calculated according to Equation (1):

(1) $$Q=900\left(I_{0}+2I_{30}+3I_{60}+\cdots +2I_{t}+\cdots +2I_{300}+2I_{330}+2I_{360}\right)$$

where *Q* represents the total electric flux (C) passing through the test piece, $I_{0}$ represents the initial current (A) and It represents the current (A) at time t (min), accurate to 0.001A.

(5) The calculated total electric flux passing through the test piece was converted into the test piece with a diameter of 95mm by the ratio of the cross-sectional area of the actual test piece.

(2) $$Q_{s}=Q_{x}{\left(\frac{95}{x}\right)}^{2}$$

where $Q_{s}$ represents the total electric flux (C) passing through the specimen with a diameter of 95 mm and $Q_{x}$ represents the electrical flux (C) passing through the specimen with diameter *x* (mm).

#### 2.2.2. Effect of Sulfate Attack on Coating Treated Mortar Matrix

The coated mortar samples and the blank group were soaked in a 5% sodium sulfate solution. The soaking methods were full immersion and dry-wet cycle.

The sulfate resistance test was conducted according to Chapter 14 “sulfate resistance test” in GB/t50082-2009 “test methods for long term performance and durability of ordinary concrete”. The size of the test piece was 40 × 40 × 160 mm. The bottom surface of the test block was painted with protective coating as the test surface, and the other surfaces were sealed with epoxy resin sealant. The samples were soaked in 5% sodium sulfate solution and the test operated during a 28-day curing period. The samples were taken out from the curing room, and then their mass was measured as m0. The samples were soaked in sodium silicate solution until the set soaking period and then the mass of them was measured as m1 after wiped dry. The rate of mass change was calculated according to Equation (3). Finally, the strength of samples was measured and compared with that of the standard curing control group.

(3) $$W_{x}=\frac{m_{1}-m_{0}}{m_{0}}$$

where $m_{1}$ represents the mass after soaking (g), $m_{0}$ represents the mass before soaking (g) and $w_{x}$ represents the mass loss rate (%).

The experiment of sulfate dry–wet cycle was carried out according to the following steps:

(1) The samples were taken out in the first 2 days of the 28 days age, and then put in the oven at (80 ± 5) ℃ for 48 h. After cooling to the room temperature, they were put into a specimen box. The adjacent spacing between test pieces shall be >20 mm.

(2) The time of each cycle for soaking was (15 ± 0.5) h, 1 h for air drying, 6 h for drying at (80 ± 5) ℃ and 2 h for cooling to room temperature. The total time of each dry–wet cycle was (24 ± 2) H.

(3) When the designed number of cycles was reached, the compressive strength test was carried out in time. The compression and corrosion resistance coefficient of the sample was calculated according to Formula (4).

(4) $$K_{f}=\frac{f_{cn}}{f_{c0}}\times 100$$

where $K_{f}$ represents the compression and corrosion resistance coefficient (%), $f_{cn}$ represents the measured value of compressive strength of a group of specimens corroded by sulfate after N cycles of drying and wetting (MPA) and $f_{c0}$ represents the measured value of compressive strength of a group of comparison specimens with standard curing at the same age as the specimens corroded by sulfate (MPA), accurate to 0.1 MPa.

#### 2.2.3. Study on the Effect of Coating Treatment on Chloride Ion Penetration Resistance of Concrete

The bottom surfaces of C30 concrete impermeability test blocks were treated with PC2, NC1, NC2, OL1 coating and LC1 control coating. After curing for 7 days, the chloride ion flux of the samples was measured by the electric flux method.

### 2.3. Microscopic Test

#### SEM Characterization & Testing

The surface blocks of SEM test samples were treated with blank samples and coating after the mortar strength test, and then the samples were soaked in absolute ethanol for 24 h to terminate their hydration. The test instrument was a German Zeiss evo-18 scanning electron microscope.

### 2.4. XRD Characterization & Testing

In this paper, XRD was used to characterize the changes in phase composition of cement-based materials after coating treatment. Characterization samples take part of the samples used for SEM characterization. After the hydration is terminated, grind them into powder with particle size less than 0.15 mm with a grinding bowl. The changes of cement hydration products before and after the coating are measured by German D8 advance X-ray diffractometer.

### 2.5. Mercury Injection Test

At present, the most widely used test method for pore analysis is mercury intrusion porosimetry (MIP). The diameter of the pores and the volume of pores of different sizes were calculated according to the functional relationship between the amount of mercury pressed into the porous system and the applied pressure. The sample used for pore analysis is a block of 3 mm thick on the surface of the coated mortar sample with a diameter of about 3–5 mm. At the age of 28 days, the hydration of the sample is terminated with absolute ethanol and dried to a constant weight in a vacuum drying oven at 105 ℃. After taking it out, it is put into a sealed bag. The porosity of the sample is measured by AutoPore IV 95 automatic mercury porosimeter, and the maximum mercury pressure is 60,000 psi.

## 3. Results and Discussion

### 3.1. Penetration Depth of Free Chloride Ion in a Coated Mortar Matrix

**Figure 3:**
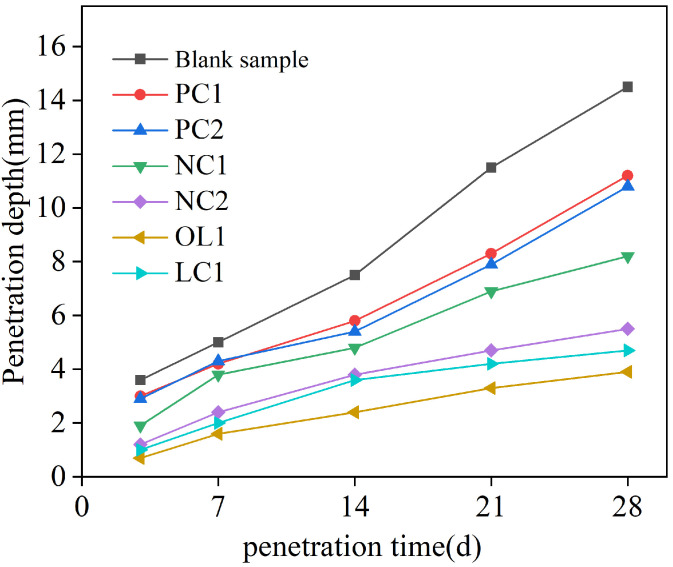
Free chloride penetration depth of coating-treated sample.

The penetration depth of a chloride ion is shown in Figure 3. In the process of chloride ion erosion, the chloride ion in the solution entered into the test block through adsorption and diffusion based on Fick’s second law [34]:

(5) $$J=-D\cdot \frac{\partial C}{\partial x}$$

where *D* represents the diffusion coefficient, *C* refers to the diffusion concentration (The movable chloride ion in cement base is free chloride ion) and *X* is the diffusion path. *∂C*/*∂X* represents the concentration gradient.

According to Fick’s second law of Equation (5), the main factors affecting chloride ion penetration were the compactness of mortar and chloride ion diffusion concentration (i.e., free chloride ion concentration in the mortar or cement-based materials). Figure 3 shows that PC1 and PC2 coatings could delay the penetration rate of free chloride ions in the mortar matrix. This is mainly due to the penetration of inorganic silicate components into the surface of the mortar matrix, having complex physical and chemical reactions with free calcium ions in cement-based materials to form hydrated calcium silicate (C-S-H) gel [23], which improves the density of the surface of mortar matrix, thereby prolonging the permeation path of chloride ions, leading to a decrease in the chloride ion concentration gradient inside the mortar, and slowing down the diffusion rate of free chloride ions in the coated mortar samples. The nano-${\mathrm{SiO}}_{2}$ in nano-modified NC1 coating could play a synergistic role with silicate components, promoting hydration and generating more C-S-H gel, to further delay the diffusion rate of chloride ions. The penetration depth after 28 days of NC1 and NC2 coating treatment sample was reduced by 43.44% and 63.07%, respectively, compared with the blank sample, indicating a considerable improvement in the anti-chloride ion penetration ability. For the organic–inorganic modified OL1 and LC1 coatings, the penetration depth of chloride ions after 28 days is 73.03% and 63.83% lower than that in the blank group, demonstrating that both coatings have the greatest influence on the chloride penetration resistance of the mortar matrix. Moreover, more organic coatings could solve the problem of coating substrate cracking caused by uneven shrinkage, which makes them a very good engineering application prospect.

### 3.2. Effect of Sulfate Attack on Coating-Treated Mortar Matrix

**Figure 4:**
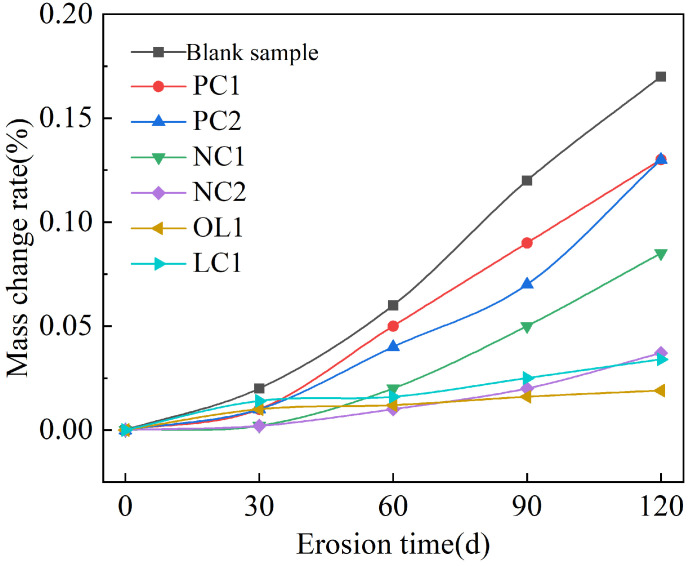
Rate of mass change of sample under full immersion.

**Figure 5:**
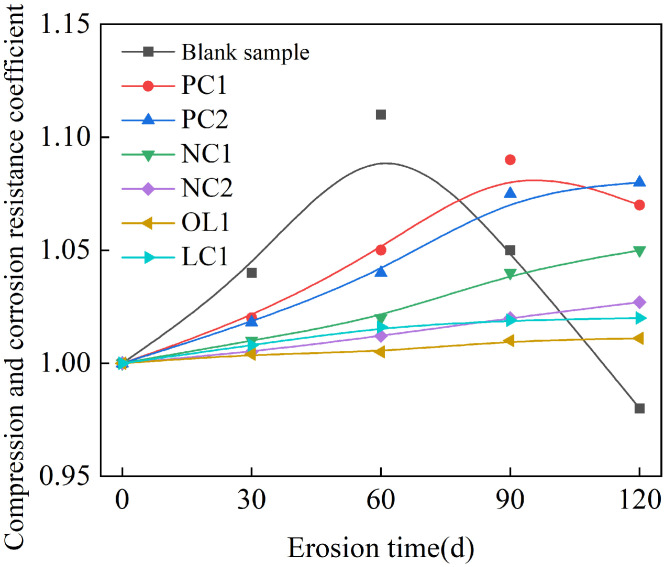
Compressive and corrosion resistance coefficient of specimens in full immersion.

**Figure 6:**
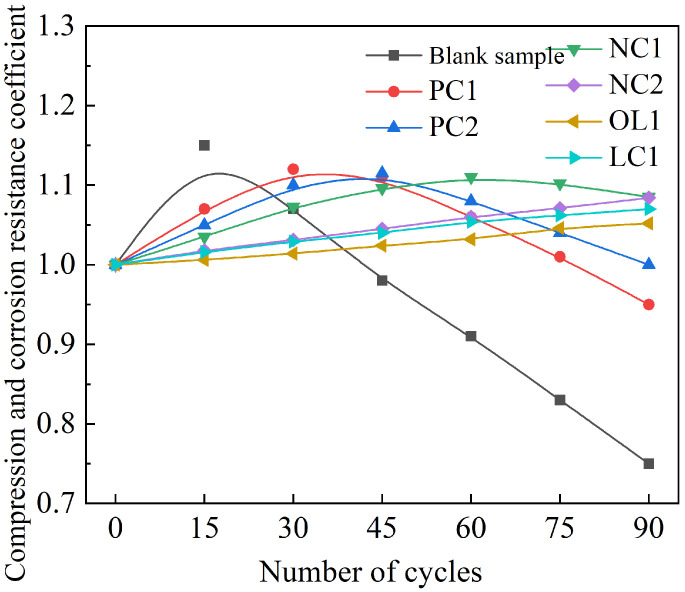
Compressive corrosion resistance coefficient of specimens under dry–wet cycle.

The rate of mass change of mortar sample under the full immersion condition is summarized in Figure 4, and the anti-corrosion coefficient of compressive strength is present in Figure 5, and the compressive corrosion resistance coefficient under the dry–wet cycle is shown in Figure 6.

Figure 4 showed that under the condition of full immersion, the mass of the mortar sample presented an increasing trend. The blank group had the largest mass growth, and its rate of mass change was 0.17% after 120 days of sulfate immersion. PC1 and PC2 coating and nano-modified NC1 coating could effectively delay the invasion of sulfate and reduce the growth rate of mortar quality through reducing the porosity of the mortar surface and water [35,36]. However, the rate of mass growth of the samples treated with NC2, OL1 and LC1 coatings was very slow due to the dissolution of calcium hydroxide on the surface of the mortar matrix within 30 days of full immersion [37]. We can infer that the pores in the surface layer of the matrix provided a channel for the invasion of sulfate, which made sulfate gather in the pores and react with cement clinker mineral C3A and hydration product $\mathrm{Ca}{\left(\mathrm{OH}\right)}_{2}$ to form ettringite and gypsum dihydrate, thus filling the capillary pores in the mortar, resulting in the increase of the strength of the matrix at the early stage of a sulfate attack. As shown in Figure 5, the strength of the blank group increased gradually between 0-60 days after sulfate attack, and decreased after 60 days of sulfate attack, while the strength of the coated sample increased slightly after sulfate attack. The strength of samples treated with PC1 and PC2 coatings began to decrease after 90–120 days of sulfate immersion, while the strength of samples treated with NC1 and NC2 nano-coatings and OL1 and LC1 coatings increased slowly within 120 days of immersion. The sulfate corrosion rate was slowed down by reducing the amount of sulfate entering into the mortar during the immersion period, and the corrosion reaction products failed to reach the critical amount of micro-cracks in the mortar. Among these coatings, the inorganic waterproof modified NC2 coating, organic–inorganic modified OL1 coating and LC1 coating have a great impact on the sulfate resistance of the mortar matrix.

After being eroded by the sulfate dry–wet cycle condition, due to the effect of expansive erosion products such as ettringite and gypsum in the wet state, and the pressure produced by sulfate crystallization in the dry state, these damages were repeated and accumulated continuously, thus accelerating the process of sulfate erosion of mortar samples [38]. After 15 times of sulfate dry–wet cycle erosion, the compressive corrosion resistance coefficient of mortar in the blank group began to decrease, while the number of cycles increased when the sulfate dry–wet cycle erosion strength of mortar in coating treatment began to decrease. Within 90 times of dry–wet cycle erosion, the strength of PC1, PC2 and NC1 coating treatment samples began to decrease. As shown in Figure 6, the strength of NC2, OL1 and LC1 coating treatment samples kept increasing slowly. On the one hand, the inorganic component of the coating reduced the porosity of the surface layer of the matrix and delayed the rate of sulfate entering into the mortar, and the coating reaction reduced the content of $\mathrm{Ca}{\left(\mathrm{OH}\right)}_{2}$ in the matrix, which was not conducive to sulfate attack reactions, so the content of ettringite is reduced. On the other hand, waterproof modification components or organic hydrophobic components brought an excellent hydrophobic effect to the surface layer of mortar. Thereby, it was difficult for sulfate ions dissolved in water to enter the interior of the matrix. Additionally, the ettringite generated in the interior of the matrix could not absorb enough water to generate expansion stress due to the improvement of the water absorption performance of the coating. Therefore, the number of soaking times was increased when the strength of the matrix began to decrease.

### 3.3. Study on the Effect of Coating Treatment on Chloride Ion Penetration Resistance of Concrete

**Figure 7:**
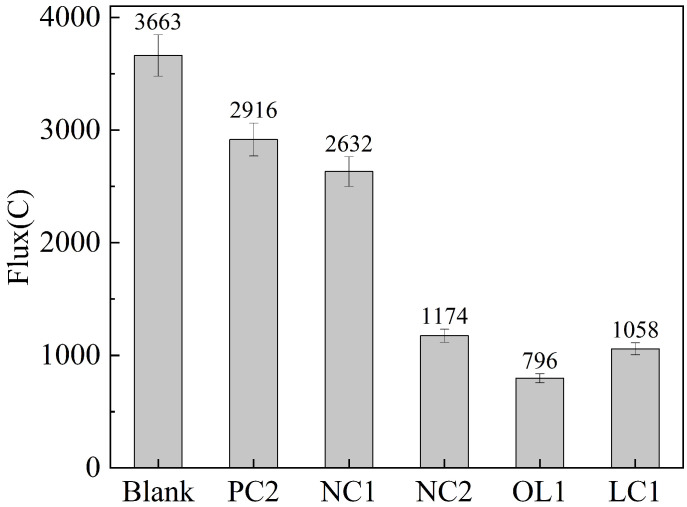
Effect of coating treatment on electric flux of concrete.

The evaluation standard of chloride ion permeability of concrete based on electric flux is present in Table 5 as ASTM C876-15 [39], and the electric flux results of C30 concrete after coating treatment are shown in Figure 7.

Figure 7 shows that the electric flux of C30 concrete blank groups were 3663 C and 3215 C, respectively, and the chloride permeability was medium. The coating treatment could reduce the chloride ion electric flux of the concrete matrix. After PC2 and NC1 coating, the chloride ion electric flux of C30 concrete samples was reduced to 2916 and 2932 C and 2612 and 2234 C, respectively, while the chloride ion permeability was still medium. After NC2 coating treatment, the electric flux of C30 concrete sample decreased to 1174C, and the C40 concrete sample electrical flux decreased to 908 C, which was less than the 1500 C required by the specification, but met the requirements of concrete corrosion resistance [40]. The effect of NC2 coating on the chloride ion permeability of concrete was similar to that of LC1 coating, which met the market requirements; After OL1 coating treatment of C30 concrete samples, the electric flux decreased to 796 C and 725 C, respectively. The chloride ion permeability of the concrete matrix decreased from medium to low, which greatly met the technical requirements of corrosion-resistant concrete for chloride ion permeability.

### 3.4. Influence of Coating Treatment on Surface Morphology of Cement-Based Materials

**Figure 8:**
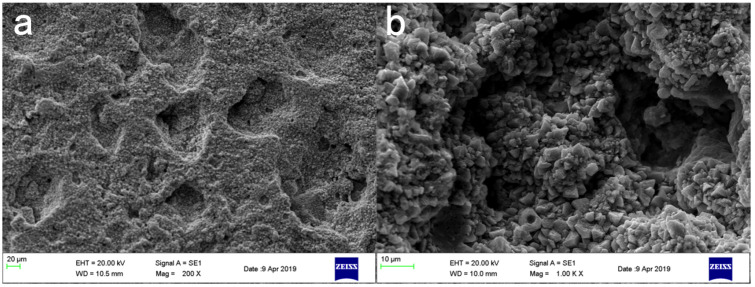
SEM figure on the surface of blank control sample. (**a**) ×200, (**b**) ×1000.

**Figure 9:**
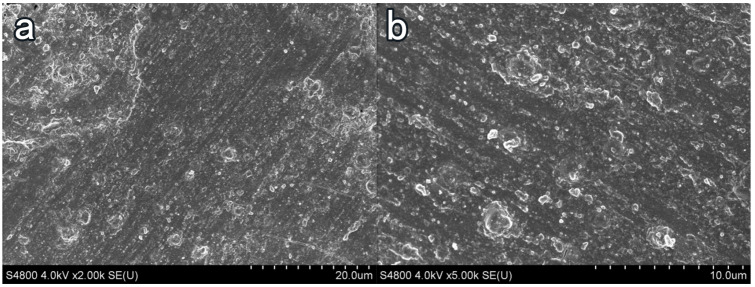
Surface treatment with NC2 coating. (**a**) ×2000, (**b**) ×5000.

**Figure 10:**
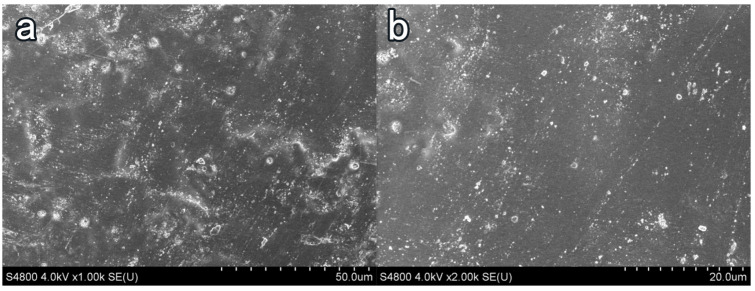
Surface treatment of OL1 coating. (**a**) ×1000, (**b**) ×2000.

**Figure 11:**
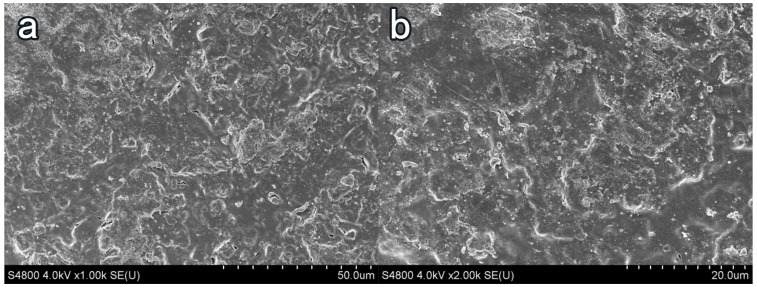
Surface treatment of LC1 coating. (**a**) ×1000, (**b**) ×2000.

In Figure 8a, the surface of the blank-cement-based sample has enormous pores and poor flatness. The hydration product $\mathrm{Ca}{\left(\mathrm{OH}\right)}_{2}$ content was high, of which the crystal structure was angular, and the combination between crystal particles was weak. Taking NC2 coating as an example, after coating treatment, the surface smoothness and compactness of the sample increased remarkably, and the crystal structure of $\mathrm{Ca}{\left(\mathrm{OH}\right)}_{2}$ became smooth. The porosity and the number of $\mathrm{Ca}{\left(\mathrm{OH}\right)}_{2}$ crystals decreased. More crystal agglomerates were produced, and an insoluble waterproof resin film formed on the surface of the substrate [41]. Few obvious pores were observed on the surface of the sample, and the flatness had greatly increased in Figure 9.After the organic–inorganic coating treatment, SEM results of the OL1 and LC1 sample surface are present in Figure 10 and Figure 11, revealing the micromorphology of the film formed by the organic coating on the substrate surface. Compared with the blank group, the large pores on the surface became smaller, and the surface flatness had increased substantially (Figure 11). However, some micro-cracks and -pores appeared in the film layer formed by LC1 coating, which could reduce the overall effect of the coating. The OL1 coating in this paper had a higher overall sealing performance, in which it formed an extremely flat and dense film layer on the surface of the substrate (Figure 10). The white spots in Figure 10 are the pits formed by the coating filling large pores on the surface of the substrate. After coating treatment, the durability was improved and the harmful media and substances find it hard to invade the substrate.

**Figure 12:**
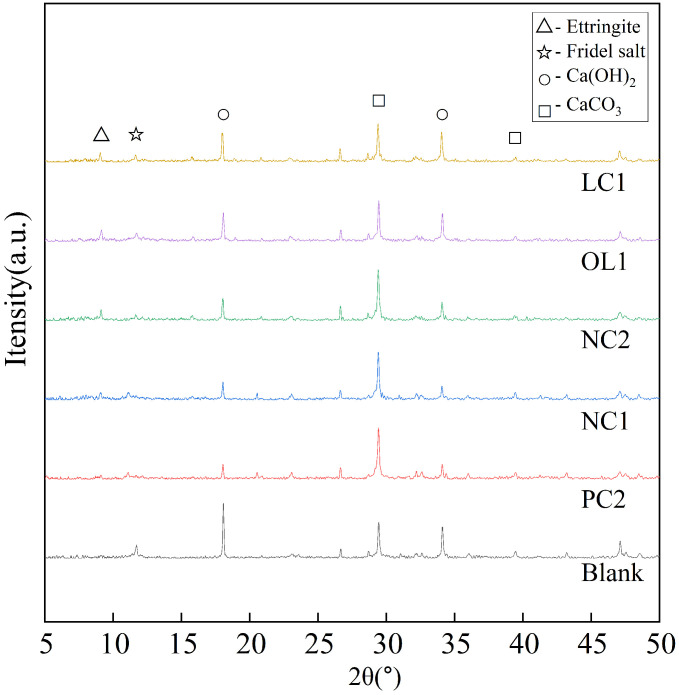
Surface treatment of LC1-coated specimen.

It can be seen from Figure 12 that the main phase groups of the blank sample are $\mathrm{Ca}{\left(\mathrm{OH}\right)}_{2}$ and C-S-H gel. $\mathrm{Ca}{\left(\mathrm{OH}\right)}_{2}$ is a layered crystal, easy to slip and weak in strength. In addition, $\mathrm{Ca}{\left(\mathrm{OH}\right)}_{2}$ is easy to precipitate after external erosion, which is the weak link of concrete. After being treated with PC2, NC1 and NC2 coatings, the diffraction peaks of each $\mathrm{Ca}{\left(\mathrm{OH}\right)}_{2}$ are weakened, while the diffraction peaks of C-S-H gel are significantly enhanced, which indicates that the coating components react with $\mathrm{Ca}{\left(\mathrm{OH}\right)}_{2}$ in the hydration products of cement-based materials and form C-S-H gel, which plays an important role in the strength of the matrix. The nano-${\mathrm{SiO}}_{2}$ particles in the nano-modified coating can not only form aggregates to act as pore filler, but moreover, because of its nanocrystalline nucleus effect, ${\mathrm{SiO}}_{2}$ can gather on its surface, which makes it react more fully with silicate components in the coating and generate more C-S-H gel. However, the diffraction peak of ${\mathrm{SiO}}_{2}$ in the XRD spectra of OL1 and LC1 treated samples is relatively weak, and the content of C-S-H gel increases less than that of other coating-treated samples. It is not difficult to find out the reasons through analysis. In the organic–inorganic coating, some inorganic components are connected with organic components by silane coupling agents, which are mainly gathered on the surface of the substrate and chemically bond with the organic coating. To make it more stable and connect with the hydration products on the surface of the matrix, the remaining inorganic components can penetrate into the interior of the matrix, and react to play a role in filling the capillary pores in the matrix.

According to the division of holes by Wu Zhongwei et al. [42], the rate of pore size distribution of the blank sample and test group sample is present in Figure 12. Compared with the blank sample, coating treatment increased harmless holes of the mortar matrix and increased its less harmful holes, harmful holes and multi harmful holes as well. After 7 days of coating reaction and curing, PC2, NC1, NC2 and OL1 coatings increased the harmless holes of mortar substrate surface by 7.67%, 9.36%, 11.71% and 21.23%, reduced the less harmful holes by 0.5%, 5.2%, 2.93% and 9.44%, the harmful holes by 16.86%, 11.9%, 25.95% and 22.15%, and the multi harmful holes by 11.78%, 5.42%, 12.05% and 20.54%, respectively. We can conclude that the organic–inorganic OL1 coating has the most obvious effect on the refinement of the pore structure on the surface of the mortar matrix; in particular, the dense film layer formed on the surface of the matrix could play a role in filling the harmful pores on the surface of the matrix and greatly reduce the infiltration of harmful media.

According to Zhou’s research [43], the most important factors determining the diffusion coefficient of diffusion media are porosity and curvature. Using a logarithmic average pore diameter instead of specimen curvature, using the product of logarithmic average pore diameter and porosity to linearly fit the diffusion coefficient, it was found that there is a high correlation between the chloride ion diffusion coefficient and the pore structure parameters of the sand slurry specimen after diffusion. The relationship between the pore distribution and ion erosion of the specimen in this paper is consistent with the laws found in the above studies.

### 3.5. Analysis of Protective Mechanism of Coating on Cement-Based Materials

According to the above analysis of the surface morphology of the coated sample SEM, it could be seen that the coating treatment would promote the two hydration products of cementitious materials, reduce the content of $\mathrm{Ca}{\left(\mathrm{OH}\right)}_{2}$ in the hydration products of cement-based materials, and produce C-S-H gel, which is conducive to strength improvement and pore refinement at the same time. The nano-component would promote the degree of reaction. The organic–inorganic coating would give full play to the respective advantages of the two components, holding the best effect on the protection of cement-based materials.

**Figure 13:**
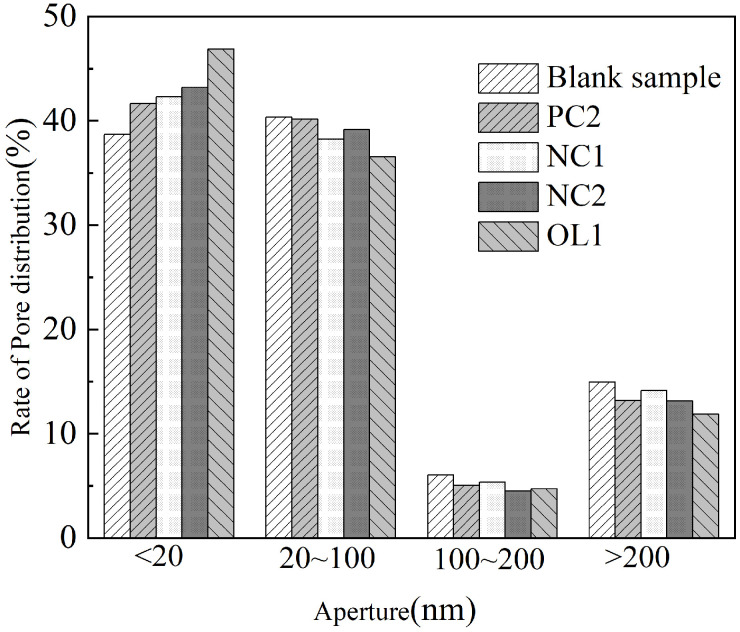
Pore size distribution of coated mortar samples.

Figure 13 presents the protective mechanism of PC2 coating on cement-based materials. PC2 coating is an inorganic permeable protective coating with lithium silicate/sodium silicate composite silicate solution as the main component. The main mechanism is that the silicate component could penetrate into the matrix through the capillary pores of cement-based materials, reacting with $\mathrm{Ca}{\left(\mathrm{OH}\right)}_{2}$ to produce $\mathrm{Ca}{\left(\mathrm{OH}\right)}_{2}$ because the cement hydration products are easy to precipitate. The newly generated C-S-H gel would fill the original pores and improve the surface density of the matrix, while the decrease of the surface capillary diameter would impede $H_{2}O$, ${\mathrm{CO}}_{2}$ and harmful carriers such as chloride and sulfate ions entering the matrix, thus enhancing the durability of the matrix.

**Figure 14:**
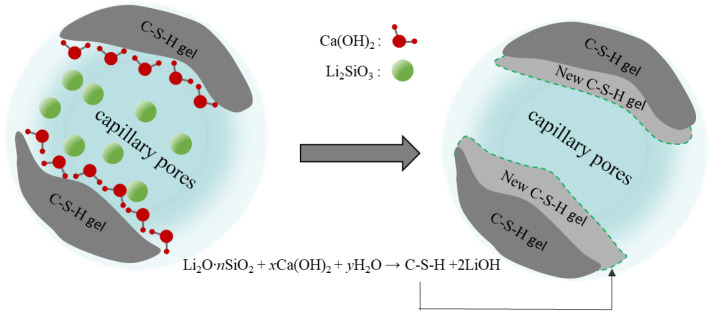
Protective mechanism diagram of PC2 coating.

Figure 14 presents the protective mechanism of PC2 coating on cement-based materials. PC2 coating is an inorganic permeable protective coating with lithium silicate/sodium silicate composite silicate solution as the main component. The main mechanism is that the silicate component could penetrate into the matrix through the capillary pores of cement-based materials, reacting with $\mathrm{Ca}{\left(\mathrm{OH}\right)}_{2}$ to produce $\mathrm{Ca}{\left(\mathrm{OH}\right)}_{2}$ becausethe cement hydration products are easy to precipitate. The newly generated C-S-H gel would fill the original pores and improve the surface density of the matrix, while the decrease of the surface capillary diameter would impede $H_{2}O$, ${\mathrm{CO}}_{2}$ and harmful carriers such as chloride and sulfate ions entering the matrix, thus enhancing the durability of the matrix.

**Figure 15:**
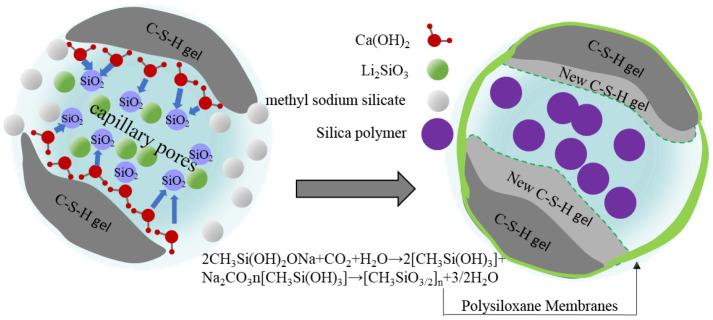
Protective mechanism of NC2 coating.

The reaction mechanism between NC2 coating and cement-based material is present in Figure 15. ${\mathrm{Li}}_{2}{\mathrm{SiO}}_{3}$ and ${\mathrm{SiO}}_{2}$ in the coating could react with $\mathrm{Ca}{\left(\mathrm{OH}\right)}_{2}$ to generate C-S-H gel. Moreover, large surface energy brought nano-${\mathrm{SiO}}_{2}$ particles’ nucleus effect, enabling $\mathrm{Ca}{\left(\mathrm{OH}\right)}_{2}$ to aggregate on their surfaces. On the one hand, it could reduce the orientation of the $\mathrm{Ca}{\left(\mathrm{OH}\right)}_{2}$, making the reaction between the coating functional components and $\mathrm{Ca}{\left(\mathrm{OH}\right)}_{2}$ more sufficient and accelerating the formation of C-S-H gel [44,45]. On the other hand, the nano-${\mathrm{SiO}}_{2}$ particles, which did not participate in the reaction could also form polymers and fill the particles with smaller pores [46], making the surface structure denser. It, thereby, has better salt erosion resistance than PC2 without nano-modification. In addition, compared with NC1 coating, after the sodium methylsilicate component in NC2 coating acts on the substrate surface, it would react with cement-based materials and form a layer of insoluble waterproof resin film with several molecular thicknesses on the substrate surface. At the same time, sodium methylsilicate is easy to be decomposed into methylsilicic acid when encountering water and ${\mathrm{CO}}_{2}$ in the air. Then, a polymethylsilyl ether with waterproof performance would be quickly polymerized to form a layer of polysiloxane film [47] on the surface of the substrate. It has strong hydrophobicity [48] since the methyl of this film is outward, which could improve the waterproof performance of cement-based materials. Therefore, it has a strong barrier effect on harmful corrosive substances with water as the medium.

**Figure 16:**
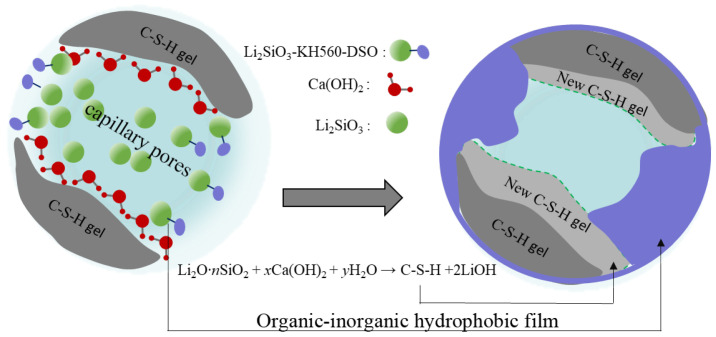
Protective mechanism of OL1 coating.

The protective mechanism of OL1 coating for cement-based materials is shown in Figure 16. ${\mathrm{Li}}_{2}{\mathrm{SiO}}_{3}$ could not only react with $\mathrm{Ca}{\left(\mathrm{OH}\right)}_{2}$ and generate C-S-H gel, but also could introduce organic segments into inorganic coatings by the condensation reaction of the surface hydroxyl groups and the silicon hydroxyl groups hydrolyzed by KH560 silane coupling agents. The organic hydrophobic component dimethylsilicone oil was connected to the non-hydrolyzable group of silane coupling agents through chemical bonding or physical winding, thus forming the molecular structure of ${\mathrm{Li}}_{2}{\mathrm{SiO}}_{3}$-kh560-dso and becoming the main component of organic–inorganic composite coating. Its organic component dso-kh560 was polymerized by shrinkage on the surface of the substrate and its large pores, and thus a film with strong hydrophobicity was formed. After curing, the film could fill the surface pores and protect the substrate surface. On the other hand, the hydrophobicity of the coating also kept the substrate closed, which could effectively block harmful corrosive media or substances in the external environment, to improve the durability of the substrate.

## 4. Conclusions

In this paper, from the perspective of the research on the surface protective materials of cement-based materials, based on the silicate permeable protective materials, the effects of several different types of coatings on the sulfate corrosion resistance and chloride ion permeability of a mortar matrix and the chloride ion permeability of a concrete matrix with different strengths were studied. The main conclusions of this study are as follows:

(1) A lithium-based coating can effectively delay the penetration rate of chloride ions and sulfates into the interior of the mortar, and nano-modified NC2 and organic–inorganic modified OL1 can further improve their salt resistance, achieving similar performance to the commercial coating LC1. However, there is a lack of quantitative analyses of the interaction mechanism between the coating and cement-based materials, which requires further testing to determine.

(2) A lithium-based coating could penetrate into the mortar, promote the formation of hydrated C-S-H gel, increase the surface density, and finally extend the penetration path of chloride ions, thus delaying the penetration rate of free chloride ions into the mortar matrix. On this basis, the nano-${\mathrm{SiO}}_{2}$ could reduce the porosity of the mortar surface, to further improve the chloride ion penetration resistance of the matrix. The hydrophobic film formed by sodium methylsilicate in NC2 coating improved the chloride ion penetration resistance of the matrix. OL1 and LC1 coatings could not only improve the compactness of the surface layer of the mortar matrix, but also form a solid and sealed hydrophobic layer on the surface layer of the mortar matrix, which greatly improved the chloride ion penetration resistance of the mortar matrix. Therefore, they have the most excellent salt erosion resistance in this group of tests. However, there is a lack of further microscopic testing for the erosion of different substances or the penetration of media, and there is a lack of detailed research on the reaction and action process of the effective components in the coating.

## Figures and Tables

**Table 1:** Technical performance index of cement.

Test Items	Technical Standard	Test Result
Flexural strength at 3 d/MPa	≥3.5	4.6
Flexural strength at 28 d/MPa	≥6.5	7.3
Flexural strength at 3 d/MPa	≥17.0	19.8
Flexural strength at 28 d/MPa	≥42.5	42.9
Initial setting time	≥45	110
Final setting time	≤390	350
Stability	Must be qualified	qualified
Fineness (80) µm sieve residue (%)	≤10.00	3.79
Specific surface area/m^2^/kg	>300	347

**Table 2:** Chemical composition of cement (wt. %).

Chemical Composition	SiO_2_	CaO	Fe_2_O_3_	Al_2_O_3_	MgO	Alkali Content	Loss on Ignition
content/%	20.56	60.96	4.89	5.56	2.93	0.5	1.64

**Table 3:** Comparison of grain size specification of “ISO standard sand” with international standard.

ISO 679:2009 [32]		China ISO Standard Sand [33]	
**Grain Size Specification (µm)**	**Cumulative Sieving Amount (%)**	**Grain Size Specification (µm)**	**Cumulative Sieving Amount (%)**
2000	0	2000	0
1600	7 ± 5	1600	7 ± 4
1000	33 ± 5	1000	33 ± 4
500	67 ± 5	500	67 ± 4
160	87 ± 5	160	87 ± 4
80	99 ± 1	80	99 ± 1

**Table 4:** Optimization of coating ratio.

Number	Lithium Silicate	Sodium Silicate	Silica Sol	Sodium Methylsilicate	Silane Coupling Agent	Methyl Silicone Oil	Helper Component
PC1	40%	—	—	—	—	—	Surfactants, pH regulators, dispersants, defoamers, film-forming AIDS, etc.
PC2	40%	10%	—	—	—	—	
NC1	40%	10%	30% × PC2	—	—	—	
NC2	40%	10%	30% × PC2	5% × PC2	—	—	
OL1	40%	10%	—	—	100% × PC2	2% × PC2	

**Table 5:** Evaluation standard of chloride ion permeability based on passing electrical charge.

Quantity of Charge q (in Coulombs)	Chloride Ion Permeability
>4000	High
2000–4000	medium
1000–2000	low
100–1000	very low
<100	could be ignored

## Data Availability

Some or all data, models, or code that support the findings of this study are available from the corresponding author upon reasonable request. All data, models, and code generated or used during the study appear in the submitted article.
